# Association between age, gender, and oral traumatic ulcerative lesions: a retrospective study

**DOI:** 10.1186/s12903-024-04312-8

**Published:** 2024-05-07

**Authors:** Qi-Lu Zou, Zhi-Qun Tang, Li-Shan Huang, Xin-Hong Wang, Zhe-Xuan Bao

**Affiliations:** 1 Department of Oral Medicine, School and Hospital of Stomatology, Guangdong Engineering Research Center of Oral Restoration and Reconstruction & Guangzhou Key Laboratory of Basic and Applied Research of Oral Regenerative Medicine, Guangzhou Medical University, Dongfeng West Road 195, Guangzhou, 510182 China; 2 Department of General Dentistry II, School and Hospital of Stomatology, Guangdong Engineering Research Center of Oral Restoration and Reconstruction & Guangzhou Key Laboratory of Basic and Applied Research of Oral Regenerative Medicine, Guangzhou Medical University, Guangzhou, China; 3 Department of Endodontology, School and Hospital of Stomatology, Guangdong Engineering Research Center of Oral Restoration and Reconstruction & Guangzhou Key Laboratory of Basic and Applied Research of Oral Regenerative Medicine, Guangzhou Medical University, Guangzhou, China

**Keywords:** Traumatic ulcerative lesions, Oral cavity, Clinical characteristics, Etiological factors, Age, Gender

## Abstract

**Background:**

Oral traumatic ulcerative lesions (OTUL) are commonly encountered in clinical practice, yet there is limited research on their clinical characteristics and traumatic etiological factors. This retrospective study aimed to analyze the age, gender, clinical characteristics, and traumatic etiological factors in a large cohort of patients with OTUL and provide valuable insights for dental clinicians to optimize patient care and prevention strategies.

**Methods:**

A total of 1543 patients with OTUL were enrolled in this study. Age, gender, medical history, clinical characteristics and traumatic etiological factors were collected and analyzed. Logistic regression analysis was performed to determine the significance of age and gender as factors related to OTUL.

**Results:**

The study revealed significant variations in clinical characteristics and traumatic etiological factors among different age groups and between genders. Logistic regression analysis demonstrated that both age and gender were significant factors related to OTUL.

**Conclusion:**

The clinical characteristics of OTUL and traumatic etiological factors appear to be significantly different according to age and gender. More targeted prevention strategies should be implemented for all age and gender groups.

## Background

Ulcerative lesions of the oral mucosa can be basically categorized into four subtypes: infection, immune related, neoplastic and traumatic [1]. Oral traumatic ulcerative lesions (OTUL), encompassing a spectrum of ulcerations or erosions within the oral mucosa attributed to the antecedent history of trauma, represent one of the most common types of ulcerative lesions encountered in clinical practice [2–6]. A previous study on 2747 patients with oral mucosa lesions showed that OTUL was diagnosed in 6.3% of cases, making it one of the most frequently diagnosed types of oral mucosa lesions [6]. A similar result was obtained from an east China study [7].

The severity and site of OTUL exhibit variation depending on the nature of the causative agent, typically arising from mechanical, thermal, and chemical traumas. Common contributors include accidental tooth bites, the presence of sharp edges on teeth or dental prosthetics, exposure to hot foods, self-inflicted behaviors, excessive tooth brushing, and iatrogenic injuries [2, 8].

The accurate diagnosis of OTUL represents a critical challenge for clinicians [1, 5]. The first reason is that ulcerative lesions of various origins have strong similarities in clinical presentation and histological features [1, 3, 5]. Second, the traumatic factors are quite complex and are easily ignored in clinical practice [1, 2]. Few studies have focused on the evaluation of OTUL; most of the existing studies had small sample sizes, and the summarized traumatic factors were not sufficiently comprehensive. The lack of studies of this condition may lead to traumatic etiological factors being overlooked by dental clinicians.

Herein, a retrospective study was used to analyze the characteristics of OTUL in a large cohort of patients in order to aid in further understanding of traumatic factors in the oral cavity and the prevention and management of traumatic ulcerative lesions of the oral mucosa.

## Methods

A retrospective cross-sectional study was conducted on patients who were diagnosed with oral traumatic ulcerative lesions (OTUL) between April 2018 and January 2023 at the department of Oral Medicine, Affiliated Stomatology Hospital of Guangzhou Medical University. The medical records from an electronic medical record system were carefully analyzed by three authors independently (QL Zhou, ZQ Tang and ZX Bao). The level of agreement between different observers was assessed using Kendall’s coefficient of concordance (Kendall’s W), with the KW value of 0.514 (*P*<0.001). The inclusion criteria were as follows: presence of a clear and documented history of trauma occurring prior to the onset of ulcerative lesions in the oral cavity; manifestation of definite ulcerative lesions consistent with a traumatic origin, supported by clinical examination and diagnostic findings; detailed description of the traumatic etiological factors documented in the medical records; confirmation that the ulcerative lesions demonstrated signs of healing within 2–4 weeks after the removal or cessation of the putative traumatic incident. Exclusion criteria included: the medical records were incomplete; the traumatic etiological factors were not clearly described; patients with other oral ulcerative diseases, including recurrent aphthous stomatitis (RAS), erosive oral lichen planus (OLP), pemphigus vulgaris (PV), mucous membrane pemphigoid (MMP), erythema multiforme (EM), bacterial, viral and fungal infections, ulcerated oral dysplasia and oral squamous cell carcinoma (OSCC). Besides, patients with oral ulcerative lesions stemming from gastrointestinal conditions (e.g., Crohn’s disease, ulcerative colitis), hematologic abnormalities (e.g., anemia, leukemia, lymphomas), rheumatologic disorders (e.g., Behçet’s syndrome), or adverse effects of drugs were also excluded from this study. Based on the inclusion and exclusion criteria, a total of 1543 patients were finally enrolled in this retrospective study. The present study was approved by the ethics committee of our hospital and informed consent was obtained from all subjects and/or their legal guardian(s).

**Fig. 1:**
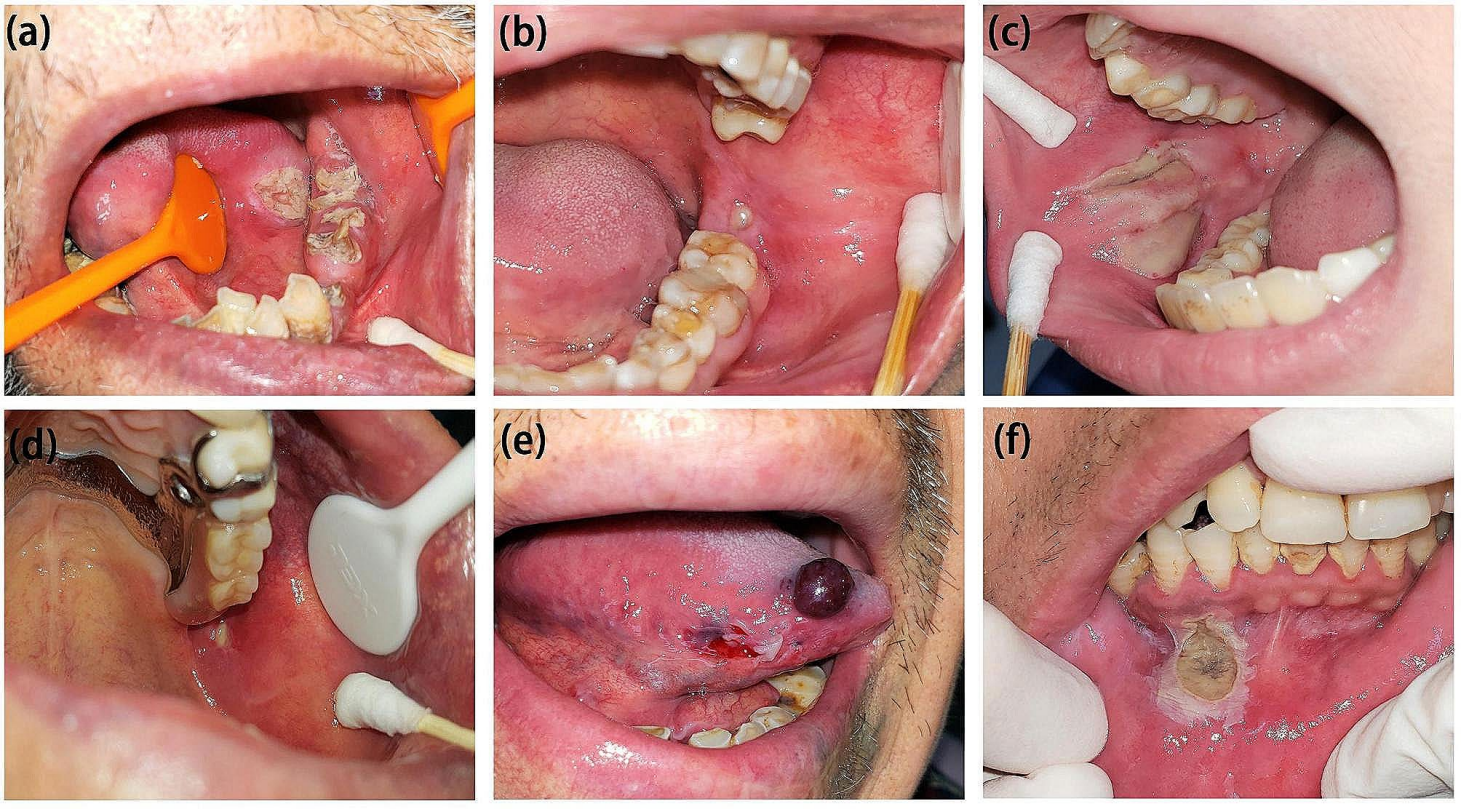
The diversity of traumatic etiological factors in oral cavity. (**a**) residual crowns or roots: A 73-year-old male patients with a huge chronic ulcer caused by lower left molar residual roots, recovered completely within 4 weeks after extraction; (**b**) third molars: A 58-year-old female patient with a chronic ulcer on the retromolar region, associated with the overerupted left maxillary third molar, the left mandibular third molar has been extracted for twenty years; (**c**) self-inflicted injuries: A 8-year-old boy has multiple histories of self-inflicted injuries and was then referred to a child psychologist; (**d**) ill-fitting removable dentures: A 70-year-old male patients with a overextended edge of maxillary removable denture; (**e**) iatrogenic injury: A 60-year-old male patient with traumatic ulcer and blood blister on the tongue attributed to laryngoscopy; (**f**) chemical irritation: A 41-year-old male patient with an ulcerative lesion resulting from a disinfectant with unknown composition and concentration

Demographic data, medical history, clinical findings and traumatic etiological factors were collected. Based on the stages of human growth, development and aging, the patients were divided into eight subgroups: 0–5 years; 6–12 years; 13–18 years; 19–39 years; 40–64 years; 65–74 years; 75–84 years and 85 years of age or above. According to the duration of ulcerative lesions, the cases were classified as acute (less than 2 weeks) or chronic (2 weeks or longer). Based on the severity of pain caused by the ulcerative lesions, participants were also classified into two grades: Grade I, no pain or only mild discomfort; Grade II, obvious pain. The locations of traumatic lesions in the oral cavity were classified as follows: upper and lower labial mucosa and vermilion, buccal mucosa, soft and hard palatal mucosa, gingiva and alveolar mucosa, floor of the mouth, retromolar area, and tongue. The traumatic etiological factors were classified into 12 types: inadvertent bite during chewing, accidental abrasion or knock, thermal injury, chemical irritation, iatrogenic injury, impacted, malpositioned, or elongated third molars, residual crowns or roots, sharp teeth and tooth edges, self-inflicted injuries, ill-fitting removable dentures, orthodontic brackets or dental retainers, and malocclusion (Fig. 1).

### Data analysis

Statistical analysis was carried out using SPSS software, version 22.0 (SPSS Inc., Chicago, IL, USA). The quantitative data were described by mean ± standard deviation and the qualitative data by rates or percentages. The comparison of percentages between groups was performed using the chi-square test, and Fisher’s exact test was applied if the observed frequency was less than 5. Spearman’s correlation coefficient was applied to evaluate the correlation between pain intensity and lesion sites. Logistic regression analysis was conducted to assess whether the age and gender were significant factors related to OTUL. A *P*-value less than 0.05 was considered statistically significant.

## Results

### Demographic characteristics

Of the 1543 patients, 759 (49.19%) were male, while 784 (50.81%) were female, with a male: female ratio of 0.96 (Table 1). The mean age (± standard deviation) of all participants was 38.91 ± 23.73 (range between 3 months and 97 years). The number and gender composition of each age group is seen in Table 1. There were significant differences in gender composition between age subgroups (*P* < 0.001).

**Table 1:** The number and gender composition of each age subgroup

Age subgroups (years)	Male *n* (%)	Female *n* (%)	Total
0–5	45 (55.56%)	36 (44.44%)	81
6–12	132 (70.59%)	55 (29.41%)	187
13–18	18 (48.65%)	19 (51.35%)	37
19–39	276 (46.7%)	315 (53.3%)	591
40–64	156 (45.88%)	184 (54.12%)	340
65–74	75 (43.1%)	99 (56.9)	174
75–84	38 (40.86%)	55 (59.14%)	93
≥ 85	19 (47.50%)	21 (52.50%)	40
Total	759 (49.19%)	784 (50.81%)	1543

### The duration and symptoms

Among all patients, 69.35% (1070/1543) had acute lesions, and the remaining 30.65% (473/1543) had ulcerative lesions that had lasted more than two weeks (Table 2). As for severity of pain, it was worth noting that a significantly higher proportion of grade I was found in the chronic group when compared with the acute group (*P* < 0.001, Table 2).

**Table 2:** The comparison of the pain severity between acute and chronic group

Duration	The Severity of Pain		Total	χ²	*P*
	Grade I *n (%)*	Grade II *n (%)*			
Acute	93 (8.69)	977 (91.31)	1070	157.397	< 0.001
Chronic	163 (34.46)	310 (65.54)	473		

When considering the age and gender, the severity of pain changed significantly (*P* < 0.001, *P* = 0.013, respectively, Table 3). Approximately 30% of patients aged 6 to 18 years and those aged 85 years and above reported either no pain or only mild discomfort (Table 3). Moreover, both the differences of gender composition ratio between acute and chronic group, and between grade I and II group were statistically significant (*P* = 0.043, 0.013, respectively, Table 3).

**Table 3:** Significant variations of clinical findings in OTUL patients with age and gender

Clinical findings		Age groups (number)								*P*	Gender		*P*
		0–5 *n (%)*	6–12 *n (%)*	13–18 *n (%)*	19–39 *n (%)*	40–64 *n (%)*	65–74 *n (%)*	75–84 *n (%)*	≥ 85 *n (%)*		Male *n (%)*	Female *n (%)*	
Duration	Acute	64 (79.01)	99 (52.94)	20 (54.05)	474 (80.20)	237 (69.71)	108 (62.07)	43 (46.24)	25 (62.50)	< 0.001	508 (66.93)	562 (71.68)	0.043
	Chronic	17 (20.99)	88 (47.06)	17 (45.95)	117 (19.80)	103 (30.29)	66 (37.93)	50 (53.76)	15 (37.50)		251 (33.07)	222 (28.32)	
The severity of pain	Grade I	19 (23.46)	64 (34.22)	10 (27.03)	67 (11.34)	46 (13.53)	25 (14.37)	14 (15.05)	11 (27.50)	< 0.001	144 (18.97)	112 (14.29)	0.013
	Grade II	62 (76.54)	123 (65.78)	27 (72.97)	524 (88.66)	294 (86.47)	149 (85.63)	79 (84.95)	29 (72.50)		615 (81.03)	672 (85.71)	
The lesion sites	Labial mucosa	26 (32.10)	30 (16.04)	6 (16.22)	35 (5.92)	14 (4.12)	11 (6.32)	3 (3.23)	2 (5.00)		70 (9.22)	57 (7.27)	0.004
	Buccal mucosa	21 (25.93)	56 (29.95)	12 (32.43)	246 (41.62)	104 (30.59)	50 (28.74)	15 (16.13)	11 (27.50)		244 (32.15)	271 (34.57)	
	Palatal mucosa	4 (4.94)	9 (4.81)	2 (5.41)	81 (13.71)	75 (22.06)	26 (14.94)	6 (6.45)	3 (7.50)		99 (13.04)	107 (13.65)	
	Gingiva	12 (14.81)	22 (11.76)	5 (13.51)	137 (23.18)	64 (18.82)	28 (16.09)	29 (31.18)	9 (22.50)		126 (16.60)	179 (22.83)	
	Floor of the mouth	0 (0)	0 (0)	0 (0)	10 (1.69)	13 (3.82)	10 (5.75)	4 (4.30)	2 (5.00)		17 (2.24)	22 (2.81)	
	Retromolar pad area	0 (0)	4 (2.14)	3 (8.11)	28 (4.74)	3 (0.88)	1 (0.57)	0 (0)	0 (0)		21 (2.77)	18 (2.30)	
	Tongue.	20 (24.69)	68 (36.36)	10 (27.03)	73 (12.35)	75 (22.06)	58 (33.33)	39 (41.94)	14 (35.00)		203 (26.75)	154 (19.64)	
Traumatic factors	Inadvertent bite	29 (35.80)	38 (20.32)	11 (29.73)	178 (30.12)	120 (35.29)	57 (32.76)	12 (12.90)	4 (10.00)		221 (29.12)	228 (29.08)	< 0.001
	Abrasion or knock	13 (16.05)	12 (6.42)	0 (0)	26 (4.40)	16 (4.71)	2 (1.15)	0 (0)	0 (0)		36 (4.74)	33 (4.21)	
	Thermal injury	1 (1.23)	1 (0.53)	0 (0)	26 (4.40)	24 (7.06)	8 (4.60)	1 (1.08)	0 (0)		34 (4.48)	27 (3.44)	
	Chemical irritation	0 (0)	0 (0)	0 (0)	4 (0.68)	5 (1.47)	0 (0)	1 (1.08)	1 (2.50)		4 (0.53)	7 (0.89)	
	Iatrogenic injury	8 (9.88)	15 (8.02)	4 (10.81)	22 (3.72)	19 (5.59)	7 (4.02)	1 (1.08)	0 (0)		26 (3.43)	50 (6.38)	
	Impacted, malpositioned, or elongated third molars	0 (0)	0 (0)	5 (13.51)	247 (41.79)	62 (18.24)	12 (6.90)	9 (9.68)	1 (2.50)		152 (20.03)	184 (23.47)	
	Residual crowns or roots	0 (0)	0 (0)	1 (2.70)	9 (1.52)	22 (6.47)	22 (12.64)	18 (19.35)	6 (15.00)		28 (3.69)	50 (6.38)	
	Sharp teeth and tooth edges	0 (0)	5 (2.67)	1 (2.70)	24 (4.06)	28 (8.24)	19 (10.92)	9 (9.68)	5 (12.50)		53 (6.98)	38 (4.85)	
	Self-inflicted injury	27 (33.33)	94 (50.27)	12 (32.43)	24 (4.06)	12 (3.53)	4 (2.30)	1 (1.08)	0 (0)		124 (16.34)	50 (6.38)	
	Ill-fitting removable dentures	0 (0)	0 (0)	0 (0)	0 (0)	22 (6.47)	33 (18.97)	40 (43.01)	21 (52.50)		50 (6.59)	66 (8.42)	
	Orthodontic bracket or dental retainer	0 (0)	11 (5.88)	2 (5.41)	16 (2.71)	1 (0.29)	0 (0)	0 (0)	0 (0)		8 (1.05)	22 (2.81)	
	Malocclusion	3 (3.70)	12 (6.42)	2 (5.41)	22 (3.72)	14 (4.12)	16 (9.20)	5 (5.38)	2 (5.00)		33 (4.35)	43 (5.48)	

### The sites of OTUL

The most frequently affected site in the oral cavity was the buccal mucosa (33.38%), followed by the tongue (23.14%), gingiva (19.77%), soft and hard palate (13.35%), and labial mucosa (8.23%). The least affected sites were the floor of the mouth and retromolar pad area, both of which were affected in 2.53% of cases. Forty-five patients had lesions in two or more sites simultaneously.

The composition of lesion sites exhibited statistically significant differences among different age groups (*P* < 0.001) and between male and female patients (*P* = 0.004, Table 3). The tongue was the most commonly affected site in the young (0–18) and the old (≥ 65), but in the middle stage (19–64), the buccal mucosa was most commonly affected. The labial mucosa was more prone to be affected in younger groups; the frequency decreased in the middle-aged and older groups. By contrast, the floor of the mouth was more commonly affected in the older groups. In terms of gender, the buccal mucosa was the most frequently affected site for both males and females. The Spearman’s correlation coefficient demonstrated a significant association between pain intensity and the palatal mucosa, gingiva, and tongue (all *P* < 0.001). Conversely, no significant correlation was observed between pain intensity and other sites (Table 4).

**Table 4:** Statistical analysis of the correlation between lesion sites and pain intensity

The lesion sites	Pain intensity		Spearman correlation coefficient	*P*
	Grade I	Grade II		
Labial mucosa	23	104	0.012	0.631
Buccal mucosa	97	418	0.043	0.094
Palatal mucosa	16	190	-0.093	< 0.001
Gingiva	25	280	-0.112	< 0.001
Floor of the mouth	5	34	-0.016	0.522
Retromolar pad area	5	34	-0.016	0.522
Tongue	91	266	0.131	< 0.001

We found that there were significant differences in lesion sites caused by different traumatic factors (χ²=1152.66, *P* < 0.001). For example, 70.49% (43/61) of thermal injury happened on the soft and hard palate (Table 5). By contrast, only 2.87% (5/174) of self-inflicted injuries occurred on the palate. The most common locations for self-inflicted injury were the tongue (57.47%, 100/174) and buccal mucosa (24.71%, 43/174). Moreover, the buccal mucosa was also the site most commonly affected by inadvertent bites (32.07%, 144/449), iatrogenic injury (31.58%, 24/76), impacted, malpositioned, or elongated third molars (61.01%, 205/336), orthodontic brackets or dental retainers (60.00%, 18/30) and malocclusion (34.21%, 26/76). By contrast, nearly 80% (78.21%, 61/78) of lesions caused by residual crowns or roots and 58.24% (53/91) of those caused by sharp teeth and tooth edges were on the tongue (Table 5).

**Table 5:** Composition of lesion sites and duration caused by different trauma factors

Traumatic factors	Lesion sites in the Oral cavity							Duration		Total
	Labial mucosa *n* (%)	Buccal mucosa *n* (%)	Palatal mucosa *n* (%)	Gingiva *n* (%)	Floor of the mouth *n* (%)	Retromolar pad area *n* (%)	Tongue *n* (%)	Acute *n* (%)	Chronic *n* (%)	
Inadvertent bite	37 (8.24)	144 (32.07)	111 (24.72)	70 (15.59)	12 (2.67)	4 (0.89)	85 (18.93)	402 (89.53)	47 (10.47)	449
Abrasion or knock	29 (42.03)	7 (10.14)	3 (4.35)	30 (43.48)	0 (0)	0 (0)	1 (1.45)	64 (92.75)	5 (7.25)	69
Thermal injury	3 (4.92)	3 (4.92)	43 (70.49)	4 (6.56)	1 (1.64)	1 (1.64)	12 (19.67)	61 (100.00)	0 (0)	61
Chemical irritation	3 (27.27)	1 (9.09)	5 (45.45)	2 (18.18)	0 (0)	0 (0)	1 (9.09)	5 (45.45)	6 (54.55)	11
Iatrogenic injury	10 (13.16)	24 (31.58)	13 (17.11)	22 (28.95)	4 (5.26)	1 (1.32)	8 (10.53)	62 (81.58)	14 (18.42)	76
Impacted or malpositioned third molars	0 (0)	205 (61.01)	0 (0)	84 (25.00)	1 (0.30)	27 (8.04)	20 (5.95)	239 (71.13)	97 (28.87)	336
Residual crowns or roots	2 (2.56)	13 (16.67)	0 (0)	4 (5.13)	0 (0)	0 (0)	61 (78.21)	30 (38.46)	48 (61.54)	78
Sharp teeth and tooth edges	7 (7.69)	25 (27.47)	1 (1.10)	6 (6.59)	1 (1.10)	1 (1.10)	53 (58.24)	42 (46.15)	49 (53.85)	91
Self-inflicted injury	16 (9.20)	43 (24.71)	5 (2.87)	8 (4.60)	0 (0)	4 (2.30)	100 (57.47)	50 (28.74)	124 (71.26)	174
Ill-fitting removable dentures	6 (5.17)	18 (15.52)	11 (9.48)	51 (43.97)	18 (15.52)	0 (0)	18 (15.52)	61 (52.59)	55 (47.41)	116
Orthodontic bracket or dental retainer	4 (13.33)	18 (60.00)	2 (6.67)	3 (10.00)	0 (0)	1 (3.33)	4 (13.33)	17 (56.67)	13 (43.33)	30
Malocclusion	11 (14.47)	26 (34.21)	12 (15.79)	23 (30.26)	2 (2.63)	0 (0)	2 (2.63)	50 (65.79)	26 (34.21)	76

### The traumatic etiological factors

In the present study, the most common traumatic factor was inadvertent bite during chewing (29.10%, 449/1543), followed by impacted, malpositioned, or elongated third molars (21.78%, 336/1543), self-inflicted injury (11.28%, 174/1543), ill-fitting removable dentures (7.52%, 116/1543), sharp teeth and tooth edges (5.90%, 91/1543), and residual crowns or roots (5.06%, 78/1543, Table 5). The frequencies of other factors were all below 5%. Chemical irritation was the least common, occurring in only 0.71% of cases (11/1543). The medical records of 24 participants described two or more traumatic factors simultaneously, so the totals of these numbers exceeded the total number of patients.

At different age groups, the common traumatic factors changed significantly (*P* < 0.001, Table 3). For patients aged 6–18 years old, the most common traumatic factor was self-inflicted injury (50.27% and 32.43%, respectively), followed by inadvertent bite (20.32% and 29.73%, respectively). In young adults, the most common traumatic factor changed to impacted, malpositioned, or elongated third molars (41.79%). In comparison to younger age cohorts, the incidence of ulcerative lesions attributed to ill-fitting removable dentures significantly increased among patients aged 65 years or older. Remarkably, among patients aged 75 years and above, ill-fitting removable dentures emerged as the primary etiological factor (43.01% and 52.50%, respectively, Table 3). Significant differences were also found in the traumatic factors between males and females. Noteworthy is the markedly elevated prevalence of self-inflicted injury factors among males, as compared to females, with rates of 16.34% and 6.38%, respectively (Table 3). This finding suggests a higher incidence of self-inflicted injuries in male patients with OTUL.

### Logistic regression analysis

Both age and gender were significantly correlated with the duration and pain intensity of OTUL (Table 6). With regard to the sites of lesions, age demonstrated significant associations with all sites except the gingiva. Conversely, gender exhibited no statistically significant associations with the lesion sites, with the exception of the gingiva and tongue (Table 6). Within the spectrum of traumatic factors, age demonstrated statistically significant correlations with 9 out of the 12, whereas gender exhibited significant associations with only 4 of them (Table 6). Inadvertent biting, the most common factor, demonstrates no significant correlation with either age or gender (*P* = 0.574 and *P* = 0.965, respectively). Impacted, malpositioned, or elongated third molars and self-inflicted injuries, identified as the second and third most common etiological factors respectively, show statistically significant correlations with both age and gender (Table 6).

**Table 6:** Logistic regression analysis on the correlation between the two factors (age and gender) and OTUL

		Factor	OTUL			
			*β*	OR	95%CI	*P*
Duration		Age	0.008	1.008	1.004–1.013	<0.001
		Gender	-0.266	0.766	0.615–0.954	0.017
Pain intensity		Age	-0.008	0.992	0.986–0.998	0.006
		Gender	-0.300	0.741	0.564–0.973	0.031
The lesion sites	Labial mucosa	Age	-0.29	0.971	0.962–0.981	<0.001
		Gender	-0.120	0.887	0.611–1.287	0.527
	Buccal mucosa	Age	-0.008	0.992	0.988–0.997	0.001
		Gender	0.147	0.936	0.936–1.435	0.177
	Palatal mucosa	Age	0.009	1.010	1.003–1.016	0.002
		Gender	0.006	1.006	0.749–1.353	0.966
	Gingiva	Age	0.003	1.003	0.998–1.008	0.258
		Gender	0.382	1.465	1.135–1.890	0.003
	Floor of the mouth	Age	0.031	1.031	1.017–1.046	<0.001
		Gender	0.101	1.106	0.579–2.113	0.760
	Retromolar pad area	Age	-0.025	0.975	0.959–0.991	0.002
		Gender	-0.065	0.937	0.492–1.786	0.844
	Tongue	Age	0.011	1.011	1.006–1.016	<0.001
		Gender	-0.459	0.632	0.496–0.804	<0.001
Traumatic factors	Inadvertent bite	Age	-0.001	0.999	0.994–1.003	0.574
		Gender	0.005	1.005	0.806–1.253	0.965
	Abrasion or knock	Age	-0.029	0.971	0.959–0.983	<0.001
		Gender	0.020	1.020	0.625–1.667	0.936
	Thermal injury	Age	0.011	1.011	1.000-1.022	0.041
		Gender	-0.327	0.721	0.429–1.211	0.216
	Chemical irritation	Age	0.016	1.016	0.992–1.042	0.196
		Gender	0.455	1.577	0.457–5.436	0.471
	Iatrogenic injury	Age	-0.019	0.981	0.971–0.992	0.001
		Gender	0.752	2.122	1.300-3.465	0.003
	Impacted, malpositioned, or elongated third molars	Age	-0.010	0.990	0.985–0.995	<0.001
		Gender	0.256	1.010	1.010–1.651	0.041
	Residual crowns or roots	Age	0.052	1.053	1.041–1.066	<0.001
		Gender	0.421	1.523	0.931–2.492	0.094
	Sharp teeth and tooth edges	Age	0.025	1.025	1.016–1.035	<0.001
		Gender	-0.510	0.600	0.388–0.928	0.022
	Self-inflicted injury	Age	-0.074	0.928	0.917–0.940	<0.001
		Gender	-0.865	0.421	0.290–0.610	<0.001
	Ill-fitting removable dentures	Age	0.106	1.112	1.092–1.132	<0.001
		Gender	0.033	1.034	0.656–1.630	0.886
	Orthodontic bracket or dental retainer	Age	-0.050	0.951	0.930–0.973	<0.001
		Gender	1.252	3.498	1.528–8.007	0.003
	Malocclusion	Age	0.004	1.004	0.995–1.014	0.383
		Gender	0.223	1.250	0.783–1.995	0.349

## Discussion

In the present study, we demonstrated that OTUL can occur in patients of any age and gender. It is widely recognized that oral mucosal disorders, such as OLP, oral leukoplakia (OLK), burning mouth syndrome (BMS), and RAS, exhibit significant associations with both age and gender [7, 9–11]. However, the clinical study regarding the correlation between gender, age, and OTUL is still lacking. In the present study, after analyzing 1543 patients, we found that the clinical characteristics of OTUL, including duration, pain intensity, the composition of lesion sites were all significantly correlated with age and gender. To the best of our knowledge, the present study might be the first to demonstrate that the clinical characteristics of OTUL vary significantly across age and gender groups. Comprehensive research on this condition is essential, offering valuable insights for dental clinicians.

Research on the clinical characteristics of OTUL requires a detailed investigation into its underlying pathogenic factors. Based on the duration, OTUL can be classified into acute and chronic forms [2, 5, 8]. The acute form, often resulting from accidental biting or hot food, is marked by a sudden onset, pronounced pain and short duration. It typically displays a white or yellowish central clear area with erythematous halo [2, 8]. In contrast, the chronic form, commonly associated with sharp tooth edges and ill-fitting dentures, typically presents with a gradual onset or slow progression [2]. It is characterized by a shallow or deep disruption of the epithelium, often accompanied by peripheral keratosis, and may be either symptomatic or asymptomatic [2]. However, existing studies might be somewhat limited in depth and warrant more thorough investigation [4, 9, 12–14]. Given the larger sample size, our study more comprehensively demonstrated the diversity and complexity of traumatic injuries in the oral cavity. Abnormal tooth position is acknowledged as a potential factor in periodontitis [15], yet few studies have revealed the traumatic impact of third molars on the oral mucosa. Our study demonstrated that impacted, malpositioned, or elongated third molars, which are often overlooked in clinical practice, were among the most commonly prevalent traumatic factors in the oral cavity. The necessity for extraction of third molars is still under debate [16–18]. When making the decision whether or not to remove third molars, clinicians generally give priority to the needs of orthodontic, periodontal or prosthetic treatment, and the prevention of caries and root resorption in second molars [18]. However, based on our research, we advocate that the potential traumatic consequences of third molars on the oral mucosa should not be disregarded. Prophylactic removal of impacted, malpositioned, or elongated third molars may prove beneficial in preventing mucosal trauma.

A clear definition and classification of self-inflicted injuries to the oral cavity have not been established [19–21]. In this study, we found that self-inflicted behaviors mainly referred to conscious or compulsive repetitive injuries to an existing oral ulcer or normal-appearing mucosa, which is usually achieved through biting with the teeth or friction and could seriously interfere with healing. A previous study on 19 patients showed that oral ulcerative lesions caused by self-inflicted behaviors were mainly on the lips and tongue, with only one case on the cheek [19]. After analyzing 27 literature cases with oral self-inflicted injuries, a report found that 25 of 27 cases had gingival lesions, and the gingiva was the most frequently affected site [22]. By contrast, in our present study of 174 patients having self-inflicted injuries, the tongue was the most affected site (nearly 60%), followed by the cheek (24.71%) and the lip (9.20%). The percentage affecting the gingiva was only 4.60%. Mounting evidence indicates that children and adolescents with attention deficit and hyperactivity disorder (ADHD) or subsyndromal ADHD have a significantly increased risk of self-inflicted injury [23–25]. A previous study also found that self-mutilation of the oral cavity is very common in mentally retarded children [26]. Routine inspection of the oral cavity was recommended in these children and those receiving neuroleptic and anti-epileptic drug therapy [26]. Based on our clinical experience, we suggest that it might be helpful for dental clinicians and psychologists to perform psychological assessment on patients with repetitive self-inflicted injuries. When possible, multidisciplinary collaboration should be implemented to avoid or stop self-mutilation of the oral cavity.

In addition to the chronic types, we also showed several common acute or transient types of traumas, including inadvertent bite during chewing, accidental abrasion or knock, thermal injury, chemical irritation, and iatrogenic injury. The findings of our study could be very useful for clinical practice and prevention. Nevertheless, traumatic factors might still have been underestimated. First, the clinical presentations of traumatic lesions in the oral cavity are various, including not only ulcerative lesions but also others, such as irritational fibroma and keratosis, which were not included in the present study [2]. Second, our study was performed with patients who were seeking treatment in a hospital and all patients had oral mucosal lesions as their chief complaint. More patients with milder symptoms might self-medicate instead of seeking medical care. Third, some specific forms of trauma, such as oral electrical burn resulting from sucking a live wire, were not found in this study. In the future, multi-center clinical or epidemiological studies on oral trauma should be conducted.

The large sample size allowed us to analyze traumatic factors in more detail. Recognizing that these factors may vary among individuals of different ages and genders holds significance for clinical practitioners. Self-inflicted injury emerged as the predominant traumatic factor across both children and adolescents. Furthermore, it is noteworthy that males exhibited a significant prevalence of self-inflicted injuries compared to females, with nearly 70% of observed ulcerative lesions manifesting chronically. As a result, it is imperative for dental clinicians to be especially attentive to young male patients who exhibit chronic oral ulcers, as this may indicate underlying self-injurious tendencies. With advancing age and eruption of the third molars, the most common traumatic factor in young adults changed to impacted, malpositioned, or elongated third molars, which has not been reported previously. In the adult population, ranging from 19 to 74 years of age, inadvertent biting during chewing emerged as the predominant cause of oral trauma, regardless of gender. However, other factors, including residual crowns or roots, sharp teeth and tooth edges, and ill-fitting removable dentures became increasingly more common with increasing age. This phenomenon is explicable as these traumatic elements, often associated with tooth wear and loss, are notably more frequent among older adults in contrast to young adults. Furthermore, these factors exhibit variance between genders. A recent study has recommended the implementation of gender-specific oral health literacy education to promote oral health within older adults [27]. Therefore, customizing strategies based on age and gender considerations becomes imperative for decreasing oral trauma and optimizing oral health outcomes.

Owing to the varying degrees of pain or discomfort, OTUL may impede the oral health-dependent quality of life, cause difficulty in speaking or swallowing, and hinder oral hygiene. Prompt and accurate diagnosis is of utmost importance. Thorough medical history taking and physical examination are required. Considering the limited diagnostic utility of laboratory tests in trauma cases, it is imperative for clinicians to demonstrate considerable patience in the acquisition of pertinent diagnostic information. In particular, the sequence of trauma and lesions should be determined and the lesions need to be consistent with their traumatic origin. Nevertheless, it should be noted that, even if some patients, such as children and the elderly, fail to confirm a history of trauma, the diagnosis of a traumatic lesion cannot be easily ruled out. Dental clinicians should continuously improve the understanding of the clinical characteristics of oral traumatic lesions and corresponding traumatic factors. Once the causal relationship is established, proper management should focus on the elimination of the causative factor and then enhancing the healing of the ulcerative lesion [1]. If ulcerative lesions persist after the elimination of the suspected causative factor within a reasonable time limit (2–4 weeks), a biopsy should be considered to confirm the diagnosis [1, 2].

Given the inherent weaknesses of retrospective studies, several limitations of this study need to be highlighted. First, the data collected from medical records might not fully describe the traumatic factors in the oral cavity. The traumatic lesions of some patients may represent the synergistic effect of two or more factors [1]. Since the occlusal relationship was not routinely described in the medical records of our study, we believe that trauma stemming from malocclusion may have been underestimated or potentially overlooked. Second, potential influencing factors, such as educational background, family income, systemic disorders, psychological factors and daily habits were not assessed in this study due to insufficient information available in the medical records. For future studies on oral trauma, it is essential to take these factors into consideration for a more comprehensive analysis.

## Conclusion

The present study showed the complexity and diversity of traumatic lesions in the oral cavity. Moreover, the clinical characteristics of OTUL and the traumatic etiological factors appear to be significantly different according to age and gender. Dental clinicians should improve their understanding of oral trauma and implement targeted prevention strategies for all age and gender groups.

## Data Availability

The datasets used and analyzed during the current study are available from the corresponding author on reasonable request.

## Abbreviations

BMS: Burning mouth syndrome
EM: Erythema multiforme
MMP: Mucous membrane pemphigoid
OLK: Oral leukoplakia
OLP: Oral lichen planus
OSCC: Oral squamous cell carcinoma
OTUL: Oral traumatic ulcerative lesions
PV: Pemphigus vulgaris
RAS: Recurrent aphthous stomatitis
